# Dr. John H. Rauch of Illinois

**Published:** 1886-01

**Authors:** 


### DR. JOHN H. RAUCH, OF ILLINOIS.

We make bold to say, and we say it without fear of contradiction that Dr. John H. Rauch has done more for the advancement and elevation of medical education in America, more for the science of preventive medicine, than any man in the Union. He has, by his zeal in the cause, and by the substantial fruits of his labor, placed a heavy debt of gratitude on the people and the medical profession of America—a debt which deserves recognition and requital at the hands of the American Medical Association. It is unnecessary to enumerate, or even to allude to his labors. They are too recent, too fresh in the minds of a grateful constituency. They have made an impress upon the times, and upon the literature of the times, in indellible characters, and coming generations will rise up and call him blessed. We will particularize one service, however, which alone should entitle him to the highest honor within the gift of organized American medicine. It was he, his genius unaided, which cut the Gordian knot *and solved the problem of regulating the practice of medicine.* There was no precedent, it was nobody's business, but he took it upon himself to constitute the Illinois Board of Health the judge of the respectability of medical colleges issuing diplomas! He drew a line and said: "All colleges, whose requirements to graduation fall below this line, shall be held to be not in good standing, and this Board will not recognize their diplomas." This was, indeed, "taking the bull by the horns," and he downed the bull, moreover. A test case was made in the courts, and the ruling of the Board was sustained! and this last case now becomes a precedent which is worth more to rational medicine in this country than all other court decisions besides. All honor to the clear-headed and stout-hearted hero of sanitary and educational reform.

This decision had the effect, at once, of causing a large number of medical colleges in America to raise the standard of requirements to matriculation and to graduation, and thus a great stride—an immense advance upward—was given to medical education. At the time we speak of, only eight colleges in America considered hygiene even worthy of mention. Now the majority of schools have embraced it in their regular curriculum.

The members of the American Medical Association should remember such services, and should elect Dr. Rauch to the highest position in the gift of that honorable body, as a just and merited reward.

---

